# Murine Experimental Autoimmune Encephalomyelitis Is Diminished by Treatment with the Angiogenesis Inhibitors B20-4.1.1 and Angiostatin (K1-3)

**DOI:** 10.1371/journal.pone.0089770

**Published:** 2014-02-26

**Authors:** Carolyn J. MacMillan, Carolyn D. Doucette, Jordan Warford, Suzanne J. Furlong, David W. Hoskin, Alexander S. Easton

**Affiliations:** 1 Department of Pathology, Dalhousie University, Halifax, Nova Scotia, Canada; 2 Department of Microbiology & Immunology, Dalhousie University, Halifax, Nova Scotia, Canada; 3 Department of Surgery (Neurosurgery), Dalhousie University, Halifax, Nova Scotia, Canada; University of Texas at San Antonio, United States of America

## Abstract

Angiogenesis is the formation of new blood vessels form pre-existing vasculature whose contribution to inflammatory conditions of the Central Nervous System is being studied in order to generate novel therapeutic targets. This study is the first to investigate the impact of two particular angiogenesis inhibitors on murine Experimental Autoimmune Encephalomyelitis (EAE), an inflammatory disease that mimics aspects of the human disease Multiple Sclerosis. The inhibitors were chosen to reduce angiogenesis by complimentary means. Extrinsic factors were targeted with B20-4.1.1 through its ability to bind to murine Vascular Endothelial Growth Factor (VEGF). Vascular processes connected to angiogenesis were targeted directly with K(1-3), the first three kringle domains of angiostatin. Mice treated with B20-4.1.1 and K(1-3) from onset of signs had reduced clinical scores 18–21 days after EAE induction. Both agents suppressed spinal cord angiogenesis without effect on local VEGF expression. B20-4.1.1 reduced spinal cord vascular permeability while K(1-3) had no effect. T cell infiltration into the spinal cord at day 21 was unaffected by either treatment. B20-4.1.1 reduced peripheral T cell proliferation while K(1-3) had no effect. Lymphoid cells from treated mice produced reduced levels of the T helper-17 (Th-17) cell cytokine interleukin (IL)-17 with no effect on the Th-1 cytokine interferon (IFN)-γ or Th-2 cytokine IL-4. However, when both drugs were added in vitro to naive T cells or to antigen stimulated T cells from mice with untreated EAE they had no effect on proliferation or levels of IL-17 or IFN-γ. We conclude that these angiogenesis inhibitors mitigate EAE by both suppressing spinal cord angiogenesis and reducing peripheral T cell activation.

## Introduction

Inflammatory diseases of the central nervous system (CNS) initiate a number of adaptive responses that include angiogenesis, the process by which new blood vessels are formed from pre-existing vasculature. Although angiogenesis is part of the normal response to injury if it becomes excessive or persistent then it can perpetuate inflammation and contribute to disease severity. Many factors regulate angiogenesis and include a major role for the 165 amino acid isoform of Vascular Endothelial Growth Factor-A (VEGF-A or VEGF) in humans (164 amino acids in mice). VEGF acts in concert with other mediators to promote new blood vessel formation. These mediators include angiopoietin (Ang)-1, Ang-2, Tumor necrosis factor, matrix metalloproteinases and other growth factors. Despite the multiple factors implicated in the regulation of angiogenesis, the 164/165 amino acid isoform of VEGF-A is regarded as a key orchestrator of angiogenesis in pathologic or inflammatory settings. Here, we propose strategies to inhibit VEGF that hold promise for the treatment of inflammatory disorders.

Experimental Autoimmune Encephalomyelitis (EAE) is an animal model of the inflammatory demyelinating human disease Multiple Sclerosis (MS) that is used to delineate factors involved in pathogenesis and treatment. Recent evidence has implicated angiogenesis in the pathobiology of both EAE and MS. Reports have documented angiogenesis in autopsy material from patients with MS [1], [2] and several studies of EAE have examined its role in disease progression [3]–[6]. The regulation of angiogenesis during EAE is similar to other inflammatory diseases, and includes an increase in VEGF expression [3], [4], [6]. In addition, we have recently documented the complimentary role played by Ang-1 and Ang-2 during EAE related angiogenesis [6].

The evidence for angiogenesis in both MS and EAE, and its likely contribution to the inflammatory component of these diseases, provides a rationale for studies on the therapeutic potential of angiogenesis inhibitors. Angiogenesis can be inhibited by binding key mediators such as VEGF, or by using drugs that inhibit angiogenesis by preventing vascular endothelial cells from generating new vessels through complex processes of cell detachment, endothelial proliferation, directed migration and tube formation. VEGF itself has been targeted in previous studies of EAE [4], [7]. In one study, disease scores were reduced when angiogenesis was inhibited with the VEGF receptor-2 antagonist SU5416 [4]. Recently, we described the impact of bevacizumab [7]. Bevacizumab binds with high affinity to human VEGF however its ability to bind to murine VEGF is more controversial [8]. Despite this uncertainty, bevacizumab reduced disease scores in murine EAE and suppressed spinal cord angiogenesis. During EAE, bevacizumab reduced T cell infiltration into the spinal cord and inhibited peripheral T cell responses associated with T helper (Th)-1 and Th-17 cells. Th-1 and Th-17 cells are key drivers of the autoimmune response during EAE [9].

The overall aim of this work was to examine the ability of two further angiogenesis inhibitors to modify EAE. The effects of these inhibitors during EAE have not been previously reported. In view of uncertainty over the precise target of bevacizumab during murine EAE, we used B20-4.1.1, a monoclonal antibody that binds with high affinity to both murine and human VEGF [10]. In order to compliment the action of B20-4.1.1 which inhibits angiogenesis by binding to extrinsic mediators, we also used K(1-3), the first three kringle domains of angiostatin, an angiogenesis inhibitor that directly targets vascular endothelial cells. Angiostatin is a proteolytic cleavage product of plasminogen composed of its first four kringle domains (a triple loop structure containing about 80 amino acids; [11]) that inhibits angiogenesis in a variety of contexts [12]–[18]. The first three kringle domains are commonly used instead of the complete protein, because they inhibit angiogenesis with greater potency [11]. We describe the ability of these inhibitors to reduce clinical scores during EAE and explore their mechanism of action through effects on spinal cord angiogenesis, vascular permeability, VEGF expression and T cell infiltration as well as their impact on peripheral T cell responses. This study provides evidence for the efficacy of these angiogenesis inhibitors in murine EAE and adds to the growing pre-clinical evidence base for angiogenesis as a therapeutic target in MS.

## Materials and Methods

### Ethics Statement

All animal experiments in this study were carried out in strict accordance with guidelines from the Canadian Council on Animal Care and approved by the Dalhousie University Committee on Laboratory Animals (Protocol Number: 11–127). Animal holding rooms were on a 12-hour dark/light cycle and water and food were provided *ad libitum*.

### Reagents

The peptide fragment containing amino acids 35–55 of Myelin Oligodendrocyte Glycoprotein (MOG_35–55_) was obtained from Sheldon Biotechnology (Montreal, QC, Canada). Complete Freund’s Adjuvant (CFA) containing mycobacterium tuberculosis H37RA (5 mg/ml), pertussis toxin, ovalbumin, horseradish peroxidase (type IV; HRP), decalcifying solution-lite and chemicals used for immunohistochemistry, hematoxylin and eosin staining and luxol fast blue-cresyl violet staining were obtained from Sigma-Aldrich (Oakville, ON, Canada). Immunohistochemistry on mouse spinal cord sections was performed using rabbit anti-mouse polyclonal antibodies from Abcam (Cambridge, MA, USA; product code in brackets): CD31 (ab28364) and vascular endothelial growth factor/VEGF-164 (ab46154). B20-4.1.1 was donated for research use by Genentech Inc. (South San Francisco, CA, USA). K(1-3) (recombinant human angiostatin, kringle domains 1–3) was obtained from Cedarlane (Burlington, ON, Canada). B20-4.1.1 and K(1-3) were stored at 4°C prior to use. Treatment doses were prepared by diluting each agent in sterile PBS at room temperature on the day of injection.

### Induction of Experimental Autoimmune Encephalomyelitis and Treatment Groups

C57BL/6 adult female mice (16–20 g, 6–8 weeks-of-age) were purchased from Charles River Laboratories Canada (Saint Constant, QC, Canada) and housed in the Carleton Animal Care Facility (Dalhousie University). Experimental autoimmune encephalomyelitis (EAE) was induced and scored as previously described [6], [7]. Briefly, on day 0, mice were inoculated subcutaneously (s.c. bilaterally at the base of the tail) with a 1∶1 mix of MOG_35–55_ and CFA (100 µg of each in 200 µl) combined with intraperitoneal (i.p.) pertussis toxin (PTX; 200 ng in 100 µl). I.p. PTX injections were repeated on day 2, and the MOG_35–55_ repeated on day 7 (s.c. bilaterally into the flanks). Mice received a clinical score based on the assessment of ascending paralysis. The following grading scheme was used to clinically score the animals: 0 no clinical deficits, 0.5 partially limp tail, 1 paralyzed tail, 2 beginning of walking deficit, 2.5 one hindlimb paralysed, 3 both hindlimbs paralysed, 3.5 hindlimbs paralysed with weak forelimbs, 4 bilateral hindlimb paralysis and 5 moribund. At the onset of clinical signs (scores of 0.5 or greater) the animals were placed into the following groups: Group 1, no additional treatment (EAE only); Group 2, B20-4.1.1 (5 mg/kg, i.p., repeated every 3 days until death); Group 3, K(1-3) (2 mg/kg, i.p., repeated every 3 days until death); Group 4, IgG (Sigma-Aldrich, 5 mg/kg, i.p., every 3 days until death). Additional groups were used as controls in which EAE was not induced and received injections (individually) of MOG_35–55_, pertussis toxin, ovalbumin, B20-4.1.1 and K(1-3). B20-4.1.1 and K(1-3) dosing regimens were based on work by Liang *et al.* (2006, [10]) and MacDonald *et al.* (1999, [19]), respectively. EAE mice were sacrificed 7, 14 and 21 days after induction, while controls were killed after 21 days. Immediately before death, mice were injected with type IV horseradish peroxidase (HRP, 0.35 mg/g dissolved in 100 µl saline), which circulated for 15 min as part of the protocol for permeability maps. Mice were sacrificed by an overdose of sodium pentobarbital (200 mg/kg, i.p.). Following deep anesthesia, the heart was opened and a blood sample (∼200 µl) removed to assess plasma HRP concentration. One group of mice was transcardially perfused (10 ml heparin saline followed by 10 ml 10% buffered formalin). Next, the spinal column was removed *en bloc* and post-fixed in formalin (48 h) followed by decalcifying solution (24 h). Paraffin embedded tissue was then sectioned (5 µm) for permeability maps and immunohistochemistry. One group of non-perfused EAE mice and controls were used to obtain fresh spinal cord tissue to extract mononuclear cells while a third group was used to isolate peripheral lymph nodes. Further experiments were carried out in a group of perfused mice to extract mononuclear cells from the spinal cord, as detailed below.

### Immunohistochemistry

Immunohistochemistry was performed on 5 µm sections, as previously described [6]. Briefly, formalin fixed paraffin embedded sections were deparaffinized followed by antigen retrieval in citrate buffer in a decloaking chamber (Inter Medico, Markham, ON, Canada). Protein blocking was carried out in 5% horse serum dissolved in Tris buffered saline with Tween 20 (TBST) for 2 h at 37°C. Endogenous peroxidase was blocked with dual enzyme block solution (Dako, Glostrup, Denmark). Sections were exposed to primary antibody diluted in TBST for 1 h at room temperature. The following antibody dilutions were used: CD31 (1∶50) and VEGF (1∶100). Sections were washed and incubated with a pre-diluted rabbit alkaline phosphatase polymer (Inter Medico) for 30 min at room temperature. Alkaline phosphatase was detected with the Vulcan Fast Red chromogen kit 2 (Inter Medico). Sections were washed and counterstained in Meyer’s Hematoxylin before mounting. Tissue was assessed in a blinded fashion by two observers (CM and AE). Vessel counts were performed on the basis of CD31 positive profiles. VEGF expression was assessed from axon staining in the dorsal columns of the spinal cord. In each case, 2 sections of lumbosacral spinal cord were assessed and data averaged to give a final value per mouse. Individual values were then averaged to give final data. In the case of VEGF expression at day 21 mice either showed persistently increased (high) expression or reduced (low) expression. To quantify this, an arbitrary cut-off of 40% expression was used to place mice in either the high or low expression groups.

### Permeability Maps

The method has been published previously [20], [21]. Briefly, deparaffinized 5 µm thick tissue sections or plasma samples (taken from the heart after death) were combined with diaminobenzidine (8 mg/ml) and hydrogen peroxide (0.006%) to generate a brown reaction product from the enzymatic activity of HRP. During the reaction, the sample was transilluminated with a flat field light source, and sequential images recorded every 5 s for 200 s through a stereomicroscope (Leica S6D) with a camera (Hamamatsu C4742-80-12AG, Quorum Technologies, Guelph, ON, Canada) and Firewire link to a computer using customized image analysis software (Image Hopper, Samsara Research, Dorking, UK). To generate images with increasing pixel intensity, absorption (A) was calculated from A = log(I_o_/I_t_) where I_o_ is intensity in the initial image, and I_t_ intensity in subsequent images at time t. This was then converted to a regression through the initial series of images (up to 35 s) that is proportional to HRP concentration. By knowing the HRP concentration in plasma and tissue, a permeability-surface area (PS) product can be calculated from PS = Q_r_/∫(C_pl_)*dt* where Q_r_ is the amount of HRP in the tissue and ∫(C_pl_)*dt* is the time integral concentration of HRP in plasma [22]. To calculate PS in individual regions of interest on the permeability maps of the spinal cord, a hand tool was used to outline the region, and its volume calculated from the pixel number, pixel size and tissue thickness. The PS data from each region was multiplied by this volume to calculate a PS value in ml/s/g. Multiple regions were then averaged to obtain a value per section, and data from 2 sections per animal were averaged to calculate the PS in each mouse.

### Lymph Node Isolation

Mice from each experimental group were killed 21 days after induction and axillary, brachial and inguinal lymph nodes pooled using two mice per sample. Mice were killed by cervical dislocation and were not perfused. Lymph nodes were homogenized and filtered through cell separation columns (Miltenyi Biotec, Auburn, CA, USA) to generate single cell suspensions. Erythrocytes were removed by osmotic shock. Total cell numbers were assessed with trypan blue dye exclusion, and cell viability was typically over 95%. Lymphoid cells were labeled with anti-CD4-FITC and anti-TcRβ-PE monoclonal antibodies/mAb (both from eBioscience, San Diego, CA, USA) and assessed by two color flow cytometry using a FACSCaliber flow cytometer with BD CellQuest software (version 3.3), both from BD Biosciences (Missisauga, ON, Canada). For all experiments, lymphoid cells were cultured at 37°C/5% CO_2_/95% humidity in RPMI-1640 medium (Invitrogen, Burlington, ON, Canada) supplemented with 5% heat-inactivated fetal calf serum, 100 U/ml penicillin, 100 µg/ml streptomycin, 2 mM L-glutamine and 5 mM HEPES (all from Sigma-Aldrich).

### Bone Marrow-Derived Dendritic Cell (BMDC) Generation

Murine bone marrow (BM) cells were isolated by flushing the tibias and femurs of C57BL/6 mice with a 27G1/2 PrecisionGlide needle (BD Biosciences) and 10 ml of ice cold PBS. A single cell suspension was generated by mechanical disruption using an 18G1 PrecisionGlide needle (BD Biosciences). Erythrocytes were depleted by hypo-osmotic shock. BM cells were seeded onto 6 well plates (1×10^6^ per well) and cultured for 9 days at 37°C/5% CO_2_/95% humidity in RPMI-1640 medium supplemented with 10% heat inactivated FCS, 0.1% 2-ME (Sigma-Aldrich), 20 ng/ml GM-CSF (R&D systems), 100 U/ml penicillin, 100 µg/ml streptomycin, 2 mM L-glutamine and 5 mM HEPES. On day 9, BM cell cultures were stimulated with 1 µg/ml LPS (Sigma-Aldrich) to generate mature BMDC.

### Proliferation Assay

Lymphoid cells were obtained from peripheral lymph nodes (see above) and were labeled with 2 µM Oregon Green 488 dye (Invitrogen) for 15 min at room temperature and then cultured in 96 well plates (1.5×10^5^ cells/well) and stimulated for 72 h with 7.5×10^4^ anti-CD3/anti-CD28 mAb-coated T cell expander beads (Invitrogen) or restimulated with MOG_35–55_ (25 µg/ml). Cells were labeled with anti-CD4-PE mAb (eBioscience) and proliferation analyzed by two color flow cytometry on a FACSCaliber flow cytometer with BD CellQuest software (BD Biosciences). Additional experiments were carried out by combining T cells with B20-4.1.1 (100 µg/ml), K(1-3) (5 µg/ml) or IgG control (100 µg/ml). In these experiments, the lymphoid cells were further purified into CD3^+^ T cells by passage through cell separation columns (Miltenyi Biotec). Cells were either naive T cells from wild type mice, or T cells taken from mice with untreated EAE and restimulated by culture with BMDCs (3.2×10^3^/well) and MOG_35–55_ (25 µg/ml). T cells were labeled with Oregon Green to assess proliferation. Labeling with Oregon Green was carried out prior to combining the T cells with BMDCs in order to exclude BMDCs from the analysis based on their lack of fluorescence.

### Cytokine Assays

Lymphoid cells (1×10^5^) obtained from peripheral lymph nodes (see above) were cultured in 96 well plates (200 µl/well) alone or in the presence of anti-CD3/anti-CD28 mAb-coated microbeads (5×10^4^) or MOG_35–55_ (25 µg/ml) for 24 h. Following 72 h incubation, culture supernatants were harvested and interleukin-17 (IL-17), interferon-γ (IFN-γ) and interleukin-4 (IL-4) concentrations determined by ELISA (IL-17, IL-4 kits from eBioscience, IFN-γ kit from BD Biosciences). Supernatants were also assessed for IL-17 and IFN-γ from purified T cells treated ex vivo with B20-4.1.1 and K(1-3) (see above). All assays were performed in quadruplicate.

### Extraction of Mononuclear Cells From Spinal Cord

Mice were killed by cervical dislocation and the spinal cord was removed shortly after death, by flushing the vertebral canal with PBS through an 18 gauge needle attached to a syringe. These mice were not transcardially perfused. However, to assess the effect of perfusion, a separate group of mice were anesthetized with an overdose of sodium pentobarbital and transcardially perfused with 10 ml heparin saline before removing the cord. The spinal cord was cut into small pieces and added to a solution of collagenase D (Roche, 2.5 mg/ml) and DNAse I (Sigma-Aldrich, 0.25 mg/ml) in RPMI-1640 medium, and incubated for 30 min at 37°C/5% CO2/95% humidity. Tissue was then further desiccated by aspiration through an 18 gauge needle and washed by centrifugation (500 g, 5 min). Red blood cells were removed by osmotic shock, washed (500 g, 5 min), filtered, washed again and supernatant decanted. The sample was then resuspended on a Percoll gradient composed of 6 ml 38% Percoll in conical tubes and centrifuged with no brake (1085 g, 35 min). The myelin layer was removed, and the cell pellet was resuspended in PBS, washed twice by centrifugation and finally re-suspended in flow cytometry buffer (1% BSA, 0.2% Sodium Azide in PBS). Total cell numbers were assessed with trypan blue dye exclusion and cell viability was typically over 95%. Mononuclear cells were labeled with anti-CD4-FITC and anti-TcRβ-PE mAbs and assessed by two-color flow cytometry.

### Statistical Analysis

Statistical comparisons between treatment groups were made using statistical software (GraphPad Prism, La Jolla, CA, USA). Multiple groups were compared by one way analysis of variance followed by the Bonferroni test. P<0.05 was taken as significant.

## Results

### B20-4.1.1 and K(1-3) Reduce Clinical Scores in EAE

**Figure 1:**
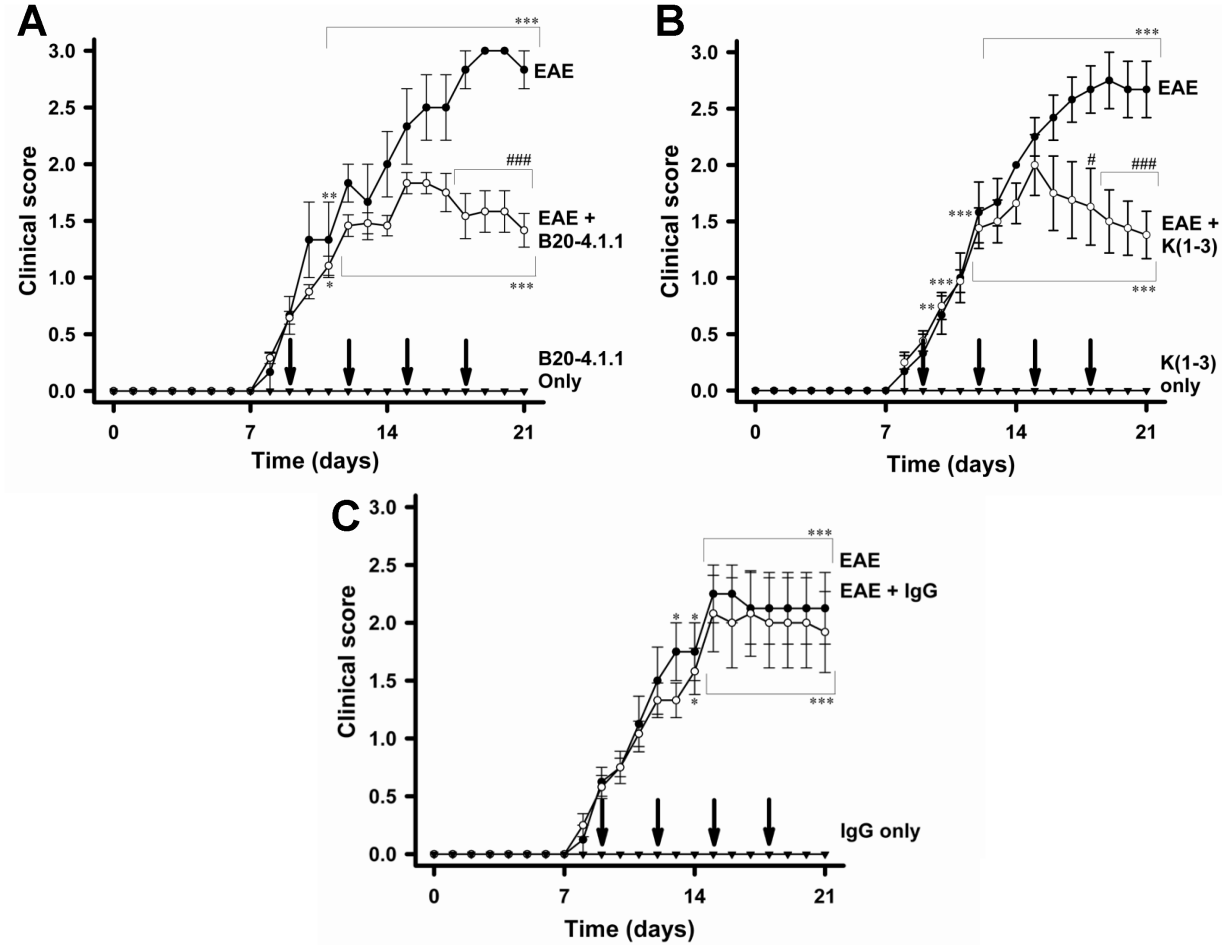
B20-4.1.1 and K(1-3) Reduce Clinical Scores in EAE. A. EAE treated with B20-4.1.1. B. Treatment with K(1-3). C. Treatment with IgG. Arrows indicate treatment days. Data shown are mean ± standard error of the mean (SEM). Indicated significance is either relative to control (*–***; P<0.05–0.001) or else compares untreated EAE with treated EAE (#,###; P<0.05, 0.001). Number of mice (n): B20-4.1.1 group: EAE only, n = 3, treated group, n = 24 up to day 14, 12 after day 14, B20-4.1.1 only, n = 3. K(1-3) group: EAE only: n = 6, treated group, n = 16 up to day 14, 8 after day 14, K(1-3) only, n = 4; IgG group: EAE only: n = 4, IgG treated, n = 12 up to day 14, 6 after day 14, IgG only, n = 2.

Both treatments were given to mice on the day of symptom onset (around day 9) and subsequently administered every 3 days until death. Fig. 1A shows the effect of B20.4.1.1 on clinical score. While treatment was started on day 9, a decline in clinical score only became apparent at day 16, with significant reductions between days 18–21 (P<0.001). K(1-3) was also administered from day 9 and like B20-4.1.1 produced a delayed reduction in clinical score, beginning at day 16 with significant reductions between days 18–21 (P<0.05–0.001, fig. 1B). Healthy controls treated with either B20.4.1.1 or K(1-3) showed no increase in clinical score. Similarly, no effect was observed in IgG controls (fig. 1C).

### B20-4.1.1 and K(1-3) Suppress Spinal Cord Angiogenesis

**Figure 2:**
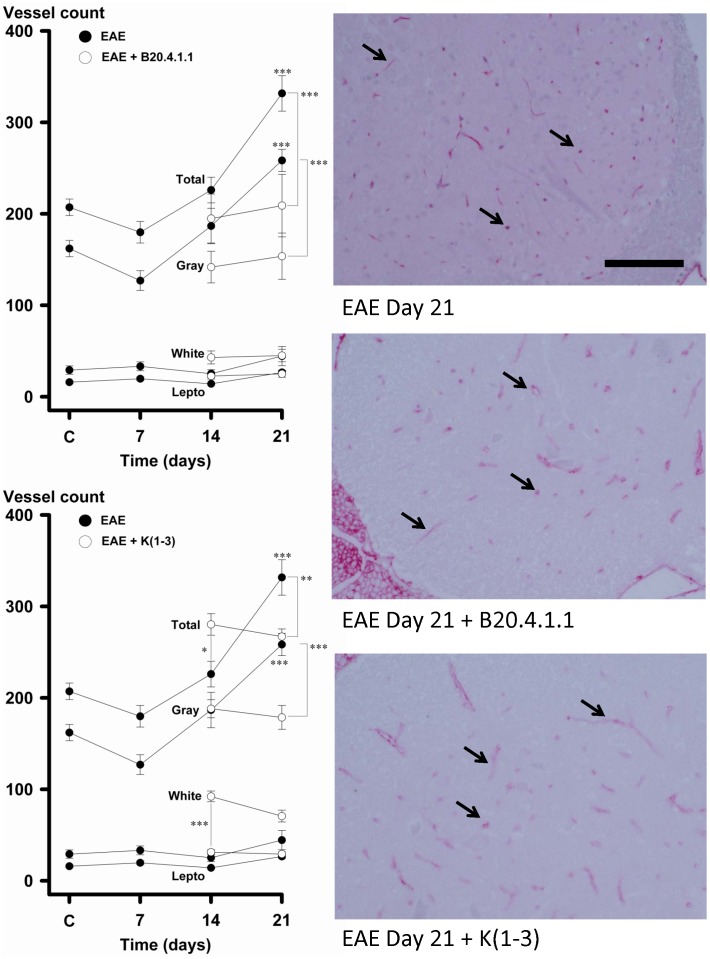
B20-4.1.1 and K(1-3) Inhibit Spinal Cord Angiogenesis During EAE. Left sided panels show vessel counts as a function of disease duration. Filled circles indicate data for untreated EAE, open circles for EAE treated with B20-4.1.1 (upper left) or K(1-3) (lower left). Data are shown as mean ± SEM obtained from per mouse values from 6-8 mice. Significance is relative to control or between points linked by cross bars (*, ***; P<0.05, 0.001). Right sided images show CD31 positive vessels in spinal cord for indicated groups. Arrows point to examples of individual blood vessels. Scale bar = 50 µm.

Angiogenesis was assessed from blood vessel counts in sections of lumbosacral spinal cord stained for CD31. Separate counts were performed in the leptomeninges, white matter and gray matter, and added to give a total vessel count. During untreated EAE, there was a significant increase in total vessel count at day 21 as well as gray matter (P<0.001), with no increase in white matter or leptomeninges (fig. 2, left sided panels, filled circles). By contrast, mice treated with B20-4.1.1 or K(1-3) during EAE showed no increase in vessel counts at day 21 (open circles). In treated mice, the total and gray matter vessel counts at day 21 were not significantly increased over controls, and significantly lower than vessel counts in untreated EAE (P<0.001). At day 14, mice treated with K(1-3) showed a significant increase in white matter vessel counts at day 14 (P<0.001) leading to a significant (P<0.05) increase in total vessel counts. This increase in white matter vessel counts had reverted to levels seen in untreated EAE by day 21. Representative images of gray matter vessel density are also shown (fig. 2, right sided panels). The vessels shown in these sections are stained red with alkaline phosphatase and include punctuate, linear and tubular forms, as indicated by arrows in fig. 2. Each of these forms, as long as they were disconnected, was counted as one blood vessel. However some individual vessels consisted of linked linear and branching forms.

### VEGF Expression is Unaffected by B20-4.1.1 or K(1-3) During EAE

**Figure 3:**
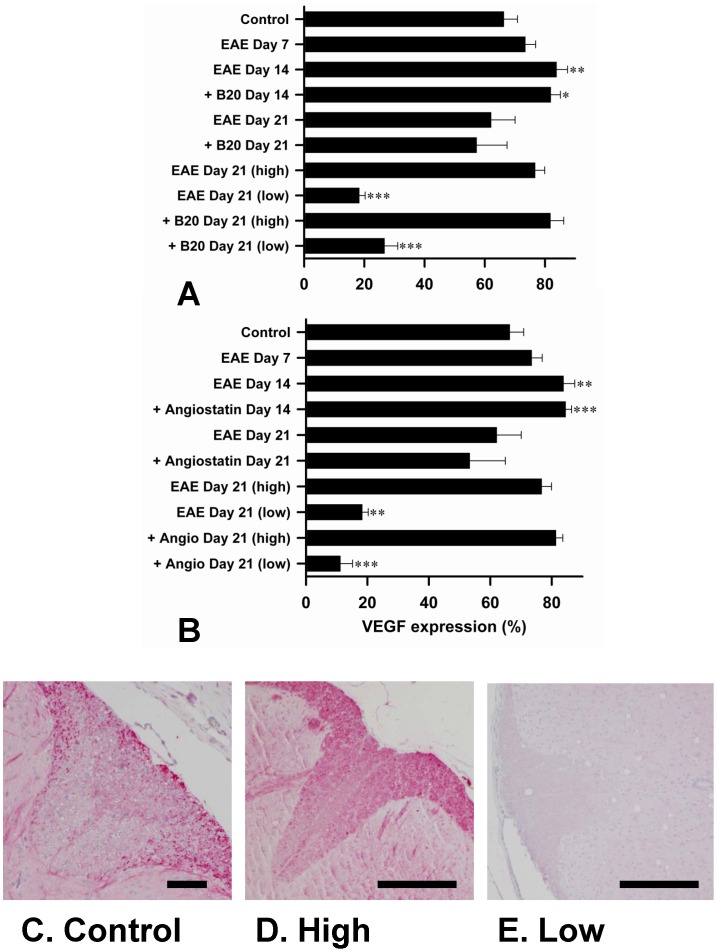
Treatment With B20.4.1.1 or K(1-3) has no Effect on VEGF Expression During EAE. A,B. Bar charts show per mouse data on VEGF expression, using the % area of positive staining in the dorsal columns. Control mice were disease-free. EAE alone is compared to mice treated during EAE with B20-4.1.1 (A) or K(1-3) (B). Day 21 data is further subdivided into high and low expression (n = 6 mice in EAE alone, n = 5 at day 21, high expression group n = 3/5, low n = 2/5; n = 9 mice for B20-4.1.1 treated mice, n = 5/9 for high expression group, low n = 4/9; n = 10 mice for K(1-3) treated mice, n = 6/10 for high expression group, low n = 4/10; data is mean ± SEM, significance relative to untreated controls, *–***, P<0.05–0.001). C–E. Representative images of dorsal column expression of VEGF for untreated controls (C), high expression B20.4.1.1 treated EAE day 21 (D) and low expression K(1-3) treated EAE day 21 (E). Scale bar = 100 µm.

**Figure 4:**
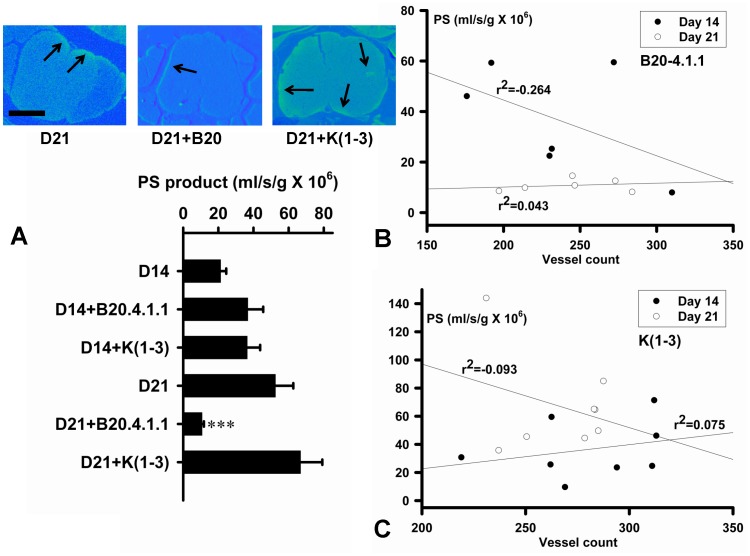
B20.4.1.1 but not K(1-3) Reduces Permeability-Surface Area Product (PS) Values During EAE. A. Upper panels show representative permeability maps for indicated treatment groups at day 21. Arrows indicate regions of increased PS within the spinal cord. Scale bar = 200 µm. Lower bar chart shows PS values at day 14 and 21 for EAE, EAE+B20.4.1.1 and EAE+K(1-3). PS values per mouse in each group (EAE only group: n = 6; B20.4.1.1 treated group, n = 6, K(1-3) treated group, n = 8); data are shown as mean ± SEM, significance compares EAE with each treatment (***; P<0.001). B, C. Regression plots for values from individual mice from day 14 (filled circles) and day 21 (open circles), comparing PS to vessel counts for mice treated with B20.4.1.1 (B) or K(1-3) (C). Regression lines through each data set are shown with the regression coefficient (r^2^).

VEGF was expressed in neurons and axons in the lumbosacral spinal cord of mice with EAE, with predominant expression in the dorsal columns, as we previously reported [6]. To quantify VEGF expression, we calculated the % area containing positively stained axons within the dorsal columns. The data show a significant increase in expression compared to untreated controls at day 14. This increase was similar whether EAE was untreated or treated with B20-4.1.1 (fig. 3A) or K(1-3) (fig. 3B). Mice with untreated EAE and each of the treatment groups showed a decline in VEGF expression at day 21; however, VEGF expression remained elevated in a subset of the mice, while others showed significant reductions below control levels. Representative images of VEGF immunostaining are shown for control mice (fig. 4C), for mice treated with B20-4.1.1 at day 21 with persistently elevated (high) expression (fig. 4D) and mice treated with K(1-3) at day 21 with markedly reduced (low) expression. If low expression is defined as dorsal column area under 40%, then 4/9 mice in the B20.4.1.1 group had low expression (18.4, 20.1, 31.4 and 36.8%) while 4 of 10 mice had low expression in the K(1-3) group (1.3, 8.4, 15.5 and 19.5%). Therefore the pattern of VEGF expression during EAE was unaltered by treatment with either agent, and does not account for their ability to suppress angiogenesis.

### Effects on Vascular Permeability

Permeability-surface area (PS) values were calculated from permeability maps of the lumbosacral spinal cord. Representative maps are shown in fig. 4A, upper panels. The permeability maps have been pseudocolored so that areas with no permeability are shown in blue, while areas of increased permeability are shown in yellow. PS values were obtained by outlining discrete regions of increased permeability on the maps. Many of these were at the margins of the spinal cord (see arrows in fig. 4A). Some spinal cord sections show a diffuse background increase in signal throughout the cord (see left and right panels in fig. 4A), however only discrete regions of permeability increase were assessed. In untreated EAE, PS values were similar at day 14 and 21 and significantly greater than healthy controls (fig. 4A, lower panel). B20-4.1.1 treatment resulted in a significant reduction in PS at day 21 (P<0.001) with no effect at day 14. K(1-3) treatment had no effect on permeability at day 14 or day 21. If the vessel surface area is estimated to be 142 cm^2^/g [23], then PS values can be divided by 142 to obtain estimates of permeability for horseradish peroxidase (HRP, the tracer used for this study). Lucifer Yellow was used to calculate permeability changes in cerebral microvessels in previous work in which values under 5×10^−6^ cm/s correspond to a stable first phase associated with mild inflammation [24]. The permeability to Lucifer Yellow (LY) can be estimated by multiplying HRP permeability by 9.82, which is the ratio of the diffusion coefficients for HRP and LY. The estimated LY permeability, P_LYest_ = 1.48±0.22×10^−6^ cm/s (n = 6) at day 14 and 3.63±0.70×10^−6^ cm/s (n = 6) at day 21 during unmodified EAE. This was reduced to 0.74±0.07×10^−6^ cm/s (n = 6) at day 21 in B20-4.1.1 treated mice (P<0.001). In previous work, we showed no relationship between permeability and vessel counts during untreated EAE [6], [7]. We investigated this relationship further in mice treated at day 14 and 21. Fig. 4B shows a negative correlation between PS and vessel counts at day 14 in mice treated with B20.4.1.1 (r^2^ = −0.264). No correlation is seen at day 21 (r^2^ = 0.043). In contrast to B20.4.1.1, fig. 4C shows no relationship between PS and vessel counts at day 14 in K(1-3) treated mice (r^2^ = 0.075) and only a weak negative correlation at day 21 (r^2^ = −0.093).

### Effect on Peripheral Lymphoid Cells

**Figure 5:**
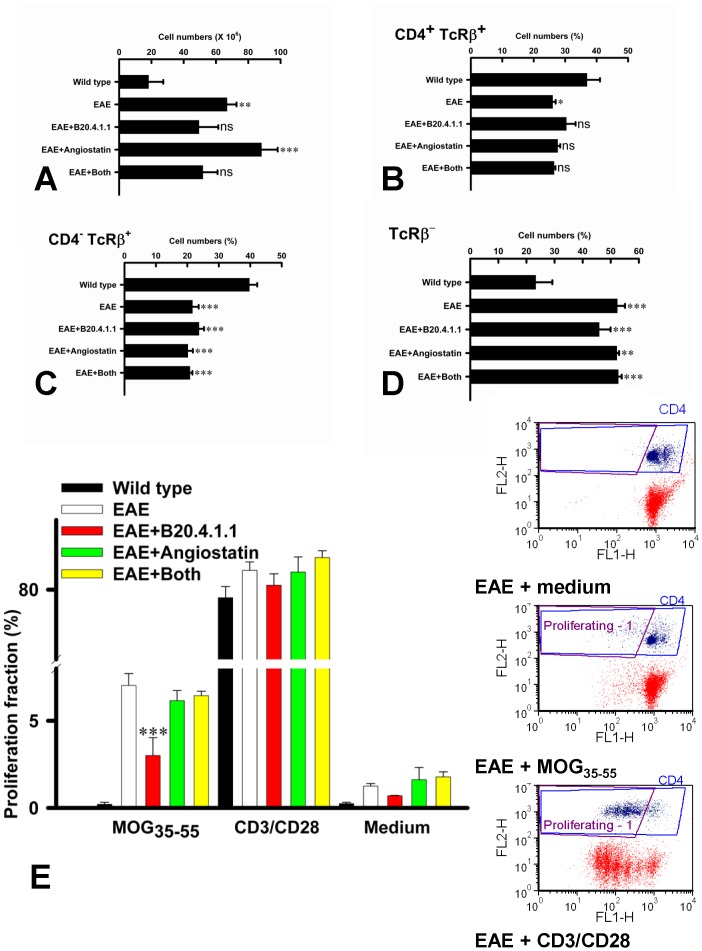
B20.4.1.1 Treatment During EAE Minimizes the Increase in Cell Numbers in Peripheral Lymph Nodes and Reduces Proliferative Responses in CD4^+^ T cells. Data are shown as mean ± SEM for wild type controls, untreated EAE, EAE+B20.4.1.1, EAE+K(1-3) and EAE+agents combined. Significance is relative to untreated EAE (ns = not significant, ***, P<0.05–0.001). A. Effect on total cell numbers (n = 3–5 samples for each group, each sample obtained by pooling lymph nodes from 2 mice). B. % of lymphoid cells co-expressing CD4 and TcRβ (n = 3–4). C. % CD4^−^TcRβ^+^ cells (n = 3–4). D. % cells lacking TcRβ (n = 3–4). E. Proliferation assay in vitro for culture medium (negative control), the inciting antigen (MOG_35–55_) and co-stimulation with T cell expander beads (CD3/CD28); n = 3–4 samples (each sample pooled from 2 mice) for each group. Representative flow cytometry plots are shown in right side panels for indicated groups. X-axis shows Oregon Green fluorescence, y-axis shows CD4 labeling. Gating for CD4^+^ cells is outlined in blue, while gating for proliferating cells is outlined in purple.

To assess the effect of B20-4.1.1 and K(1-3) on peripheral immune responses, lymph nodes were harvested at day 21 to coincide with peak disease scores. Compared to healthy (wild type) controls, there was a significant increase in lymphoid cell numbers in mice with untreated EAE (P<0.01). A similar increase was noted in mice treated with K(1-3) (P<0.001), but numbers were not significantly increased in mice treated with B20.4.1.1 or combined B20.4.1.1 and K(1-3) (fig. 5A). Flow cytometry was used to assess the proportions of different cell types among the lymphoid cells based on surface markers. Mice with untreated EAE showed a significant reduction (P<0.05) in the proportion of CD4^+^ TcRβ^+^ cells compared to controls but this was not significantly different in the treated animals (fig. 5B). All of the EAE groups (untreated or treated) had a significant reduction (P<0.001) in the proportion of non-CD4^+^ T cells (CD4^−^ TcRβ^+^, fig. 5C) with significant compensatory increases (P<0.01–0.001) in the proportion of non-T cells (TcRβ^−^, fig. 5D). Next, the fraction of proliferating CD4^+^ T cells was assessed by Oregon Green labeling. As cells proliferate, each generation contains a reduced amount of Oregon Green so that fluorescence shifts to the left. The proportion of CD4^+^ T cells in this group was used to calculate a proliferation fraction. The right panel in fig. 5E shows representative flow cytometry plots used for this calculation. The x-axis shows Oregon Green fluorescence, while the y-axis shows CD4 fluorescence. The proliferating fraction has a high expression of CD4 and contains cells whose Oregon Green fluorescence has shifted to the left upon stimulation (outlined in purple on the flow cytometry plots). The proliferation fraction (%) is the number of proliferating CD4^+^ T cells divided by the total number of CD4^+^ T cells and then multiplied by 100 to give a percent. The proliferation fraction increased when cells were treated with the inciting antigen (MOG_35–55_) and significantly blunted (P<0.001) in cells taken from mice treated with B20.4.1.1, while K(1-3) or the combination of B20.4.1.1 and K(1-3) had no effect. Proliferation was enhanced by stimulation with CD3/CD28 coated microbeads, but there were no differences between the groups (fig. 5E).

### Cell Recruitment to the Spinal Cord

**Figure 6:**
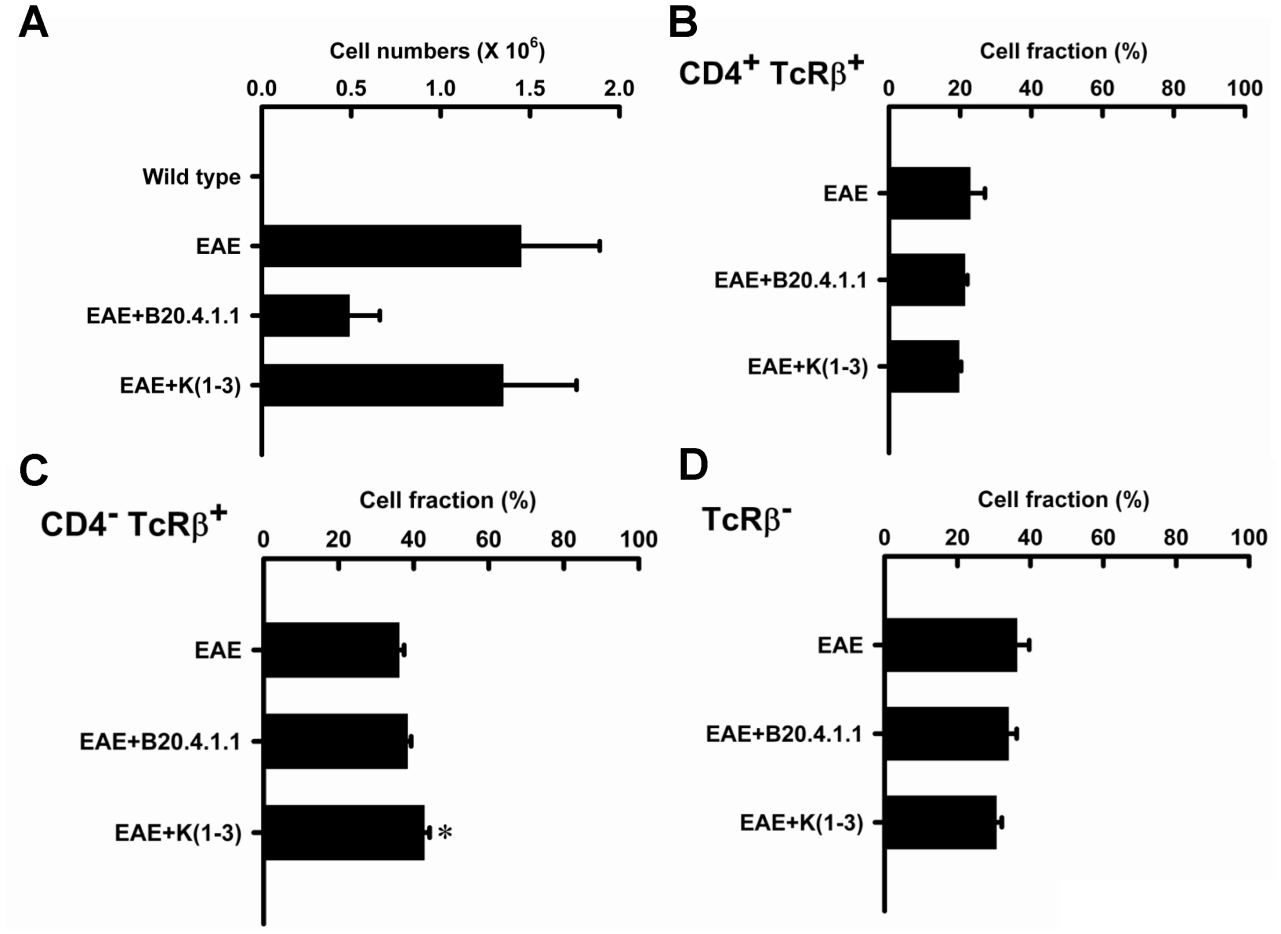
Treatment With B20-4.1.1 and K(1-3) During EAE has Little Impact on Spinal Cord Infiltration of T cells. A. Effect on total mononuclear cells isolated from spinal cord (samples pooled from 2 mice, n = 3 samples for each data set). B–D. Proportion of surface marker defined cell populations in samples from untreated EAE and treated EAE. CD4 and TcRβ status as indicated in each panel (each sample pooled from 2 mice, n = 3 samples for each data set). Data are shown as mean ± SEM, significance relative to untreated EAE (*, P<0.05).

Our previous work with bevacizumab showed a marked reduction in T cell recruitment into spinal cord [7]. To assess whether B20.4.1.1 and K(1-3) have similar effects, we isolated mononuclear cells from the spinal cord at day 21 in mice with EAE, treated or untreated. Mononuclear cells were not detected in the spinal cord of healthy wild types (fig. 6A) so further comparisons were restricted to mice with EAE. There was a significant (P<0.05) increase in the total number of spinal cord mononuclear cells in mice with EAE that was unaltered by treatment with either B20-4.1.1 or K(1-3), with no statistical differences between the groups (fig. 6A). The proportion of CD4 positive T cells (defined by co-expression of CD4 and TcRβ) was ca. 20% (based on the mean) during EAE and unaltered by treatment with either agent (fig. 6B). The proportion of CD4 negative T cells (CD4 negative TcRβ positive) cells was higher during EAE (mean ca. 36%). K(1-3) treatment resulted in a small increase to ca. 43% (fig. 6C, P<0.05) while B20.4.1.1 had no effect. The agents had no effect on the proportion of cells other than T cells (defined by negative expression of TcRβ) (fig. 6D). Fig. 6A shows that the spinal cord of non-perfused wild type control mice lack inflammatory cells. However, it is possible that EAE induces a proportion of the cells to be retained inside the vasculature. To determine this, we determined cell counts in mice with EAE who were non-perfused or perfused transcardially before removing the spinal cord. This data shows that perfusion removes about two thirds of retained cells. Perfused mice showed cell counts that were ca. 38% of those in non-perfused mice (37.73±2.42%, n = 3 where each sample pools cords from 2 mice, normalized to mean counts in the non-perfused group). Despite this reduction on perfusion, the proportion of CD4 positive and CD4 negative T cells was not altered by perfusion. The cell fraction of CD4 positive T cells for perfused animals was 4.59±0.67% compared to 8.02±2.45% for non-perfused mice. The fraction for CD4 negative T cells was 69.05±1.38% in perfused mice compared to 59.30±4.71% for non-perfused animals (n = 3, P>0.05/not significant). Therefore the cell fractions shown in fig. 6 are representative of cells within the spinal cord, but also represent the total population of pathogenic cells attracted to the cord during EAE. Overall, we conclude that the disease modifying effect of these drugs (in contrast to bevacizumab) is not due to marked reductions in cell recruitment to the spinal cord, whether adherent to vessels or within the CNS parenchyma.

### B20-4.1.1 and K(1-3) Both Inhibit IL-17 Release

**Figure 7:**
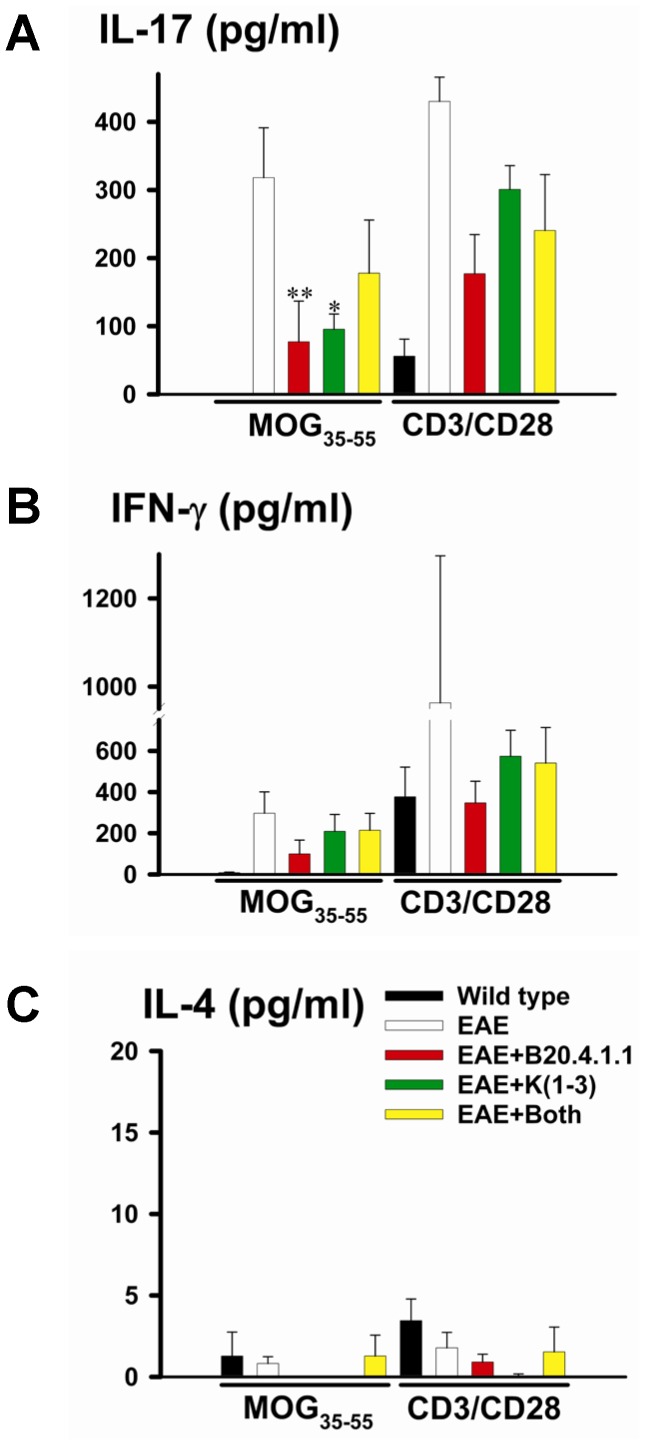
Reduced IL-17 Production in Lymphoid Cells From Mice Treated With B20-4.1.1 and K(1-3) During EAE. Data is also shown for stimulation with MOG_35–55_ as well as T cell expander beads (CD3/CD28). Supernatants were analyzed by ELISA. Bars indicate release for wild type controls, untreated EAE, EAE+B20.4.1.1, EAE+K(1-3) and EAE+treatments combined. Data are shown as mean ± SEM, each sample was pooled from 2 mice, n = 3–9 samples for each group; significance compares untreated EAE to treated groups (*–**, P<0.05–0.01). A. IL-17 release. B. IFN-γ release. C. IL-4 release.

**Figure 8:**
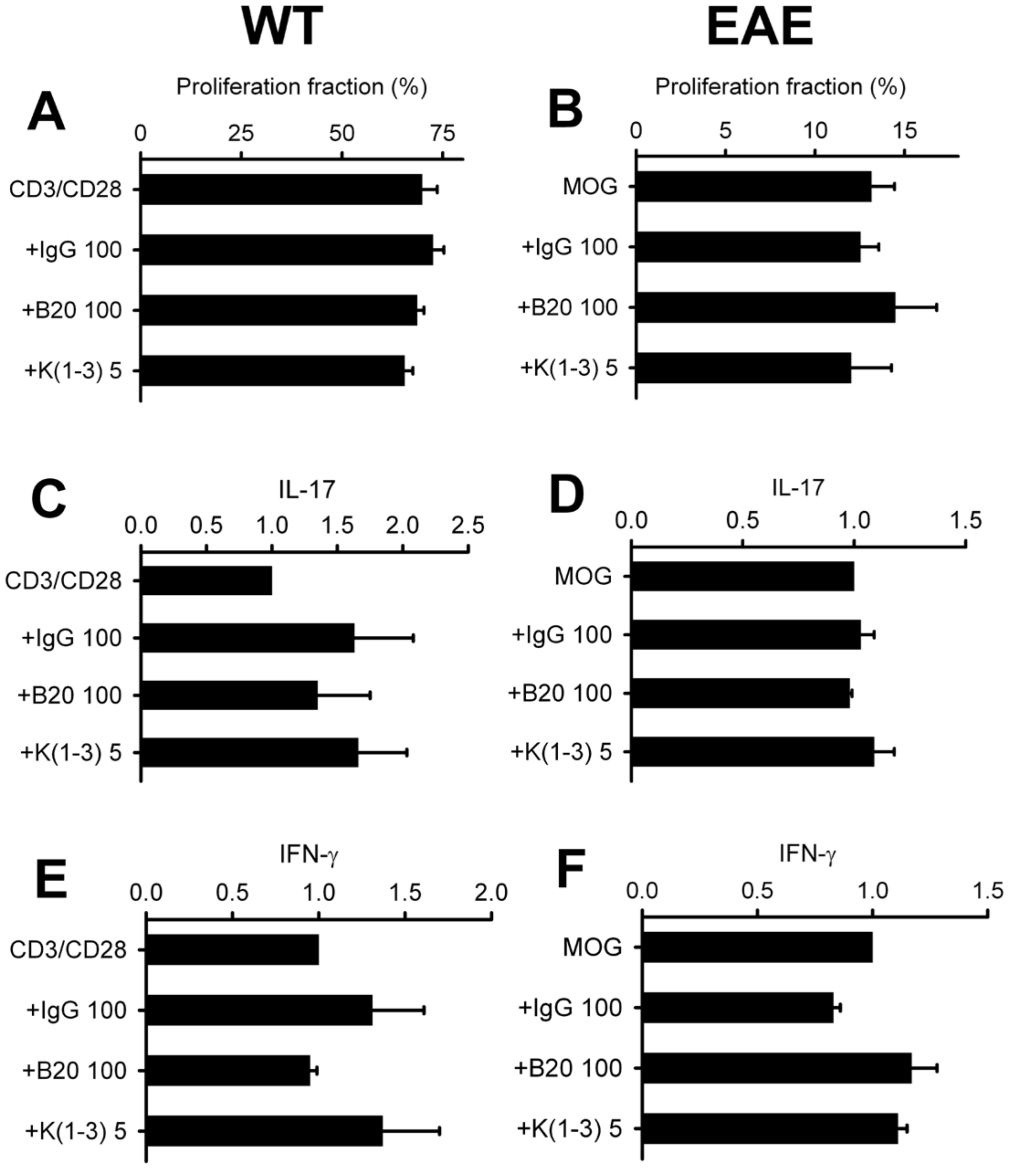
B20-4.1.1 and K(1-3) Have no Effect on Proliferation and Cytokine Secretion by Isolated T cells. A,C,E naive CD4^+^ T cells co-stimulated with T cell expander beads (CD3/CD28). B,D,F. T cells from mice with untreated EAE restimulated with MOG_35–55_ added to bone marrow derived dendritic cells. Data is normalized to untreated CD3/CD28 or MOG stimulated cells and shown as mean ± SEM, n = 3 per group; each data point pooled from 2 mice. IgG = immunoglobulin G control.

Lymphoid cells were isolated from mice with EAE and stimulated in vitro with MOG_35–55_ or CD3/CD28 coated microbeads. Supernatants were collected to measure cytokines characteristic of Th-17 cells (IL-17), Th-1 cells (IFN-γ) and Th-2 cells (IL-4). Following stimulation with MOG_35–55_, cells from mice treated with B20.4.1.1 or K(1-3) during EAE exhibited a significant reduction in IL-17 release (P<0.01 for B20.4.1.1, P<0.05 for K(1-3), fig. 7A). There were no significant effects on the release of either IFN-γ or IL-4. Significant reductions in cytokine production were not found in cells treated with CD3/CD28 coated microbeads. To assess direct effects on T cell responses, additional experiments were carried out with T cells isolated from healthy wild type mice and mice with untreated EAE, and so not exposed to either agent in vivo. The former were stimulated with CD3/CD28 coated microbeads, the latter restimulated with MOG_35–55_ added to BMDC. Cells were assessed for proliferation fraction or secretion of IL-17 and IFN-γ. In experiments where BMDC were included, these cells were not stained with Oregon Green and therefore were excluded from the analysis based on their lack of fluorescence. Addition of B20-4.1.1 (100 µg/ml) or K(1-3) (5 µg/ml) to the cultures had no effect on proliferation fraction or release of IL-17 and IFN-γ. IgG (100 µg/ml) was added to control for non-specific effects of immunoglobulin and had no effect (fig. 8).

## Discussion

### Summary of Findings

Both of the angiogenesis inhibitors used in this study, B20-4.1.1 and K(1-3), reduce clinical scores in the later stages of EAE when scores reach a peak between days 18–21 (fig. 1). As expected, both inhibitors suppressed angiogenesis in the spinal cord of mice with EAE assessed from blood vessel counts (fig. 2). Since VEGF is a key regulator of angiogenesis in this model [6], [7] we determined the impact of the drugs on VEGF expression in axons within the dorsal columns, however neither agent altered the pattern during EAE of increased VEGF expression at day 14 and reductions that begin at day 21 (6, fig. 3). The impact of both agents on vascular permeability was also explored, partly to determine the relationship between angiogenesis and permeability after drug treatment. B20-4.1.1 treatment reduced permeability at day 21, while K(1-3) had no effect (fig. 4A). There was an inverse relationship between permeability and vessel counts at day 14 in B20.4.1.1 treated mice. This was not seen with K(1-3) treatment. However the two parameters were unrelated by day 21 in both treatment groups (fig. 4B,C). Previous work suggests that angiogenesis inhibitors can have direct or indirect effects on peripheral T cell activation during EAE [7]. Therefore, we carried out studies on peripheral lymphoid cells. During EAE the number of cells in the peripheral lymph nodes is increased as a result of proliferation, and this was observed in mice treated with K(1-3). However the increase was not statistically significant in mice treated with B20.4.1.1 suggesting the possibility of reduced proliferation (fig. 5A). The proliferation fraction was indeed reduced following treatment with B20.4.1.1 but not K(1-3) (fig. 5E). Neither agent reduced T cell infiltration into the spinal cord (fig. 6); however, they both reduced levels of IL-17 produced by peripheral lymphoid cells restimulated with MOG_35–55_ (fig. 7). Although both agents had an impact on proliferation and/or cytokine profiles of lymphoid cells when administered in vivo during EAE, they had no impact on the same endpoints added ex vivo to T cells from healthy wild types stimulated with CD3/CD28 coated microbeads or to T cells from mice with untreated EAE restimulated with MOG_35–55_ (fig. 8). This implies an indirect regulation of peripheral T cell responses when EAE was treated with the two drugs, possibly related to the in vivo suppression of angiogenesis.

### Impact of B20-4.1.1 on EAE

This is the first study to report the effects of the two angiogenesis inhibitors B20-4.1.1 and K(1-3), the first three kringle domains of angiostatin, in experimental autoimmune encephalomyelitis (EAE). B20-4.1.1. belongs to a group of indirect angiogenesis inhibitors that inhibit angiogenesis through binding to exogenous pro-angiogenic factors [25]. In this case, it binds the pathogenic isoform (164 amino acids in mice, 165 in humans) of Vascular Endothelial Growth Factor-A. Previous studies have explored the role of VEGF in EAE. VEGF expression has been reported in EAE [3]–[7], [26], [27] and was found to increase before the onset of angiogenesis in several of these studies [3], . Interestingly, some studies showed decreases rather than increases in VEGF expression during EAE [5], [27]. Our work showed increases in VEGF expression before the onset of angiogenesis followed by a significant reduction after angiogenesis is established [6]. This observation may explain, at least in part, the reductions observed in previous studies. The same pattern of VEGF expression during EAE was seen in mice concurrently treated with both agents (fig. 3). In previous work, we showed that the VEGF binding monoclonal antibody bevacizumab suppresses angiogenesis and reduces clinical scores during EAE [7]. By contrast, bevacizumab had an impact on clinical scores within a few days of initiation, and several days before the onset of angiogenesis. Taken together, these effects are not just attributable to angiogenesis suppression alone. The ability of bevacizumab to block murine VEGF has been questioned [8] despite its high affinity for human VEGF [28]. The present study addresses this limitation by using B20-4.1.1, a monoclonal antibody that binds with high affinity to both murine and human VEGF [10]. The use of B20-4.1.1 in this study lays to rest the suggestion that direct binding to VEGF cannot reduce angiogenesis in murine EAE and have an impact on disease parameters. As well as the reduction in clinical scores, treatment with B20.4.1.1 suppressed angiogenesis (fig. 2) and vascular permeability (fig. 4). It also reduced proliferation and IL-17 production by peripheral lymphoid cells taken from treated mice (figs. 5,7). These effects are strikingly similar to those of bevacizumab [7], however the two agents are not identical. Treatment with B20.4.1.1 during EAE had no impact on T cell infiltration into the spinal cord at day 21 (fig. 6) despite the concurrent reduction in blood vessel density (fig. 2) which is in marked contrast to bevacizumab, where treated mice showed reduced total cell counts in spinal cord as well as reductions in the relative proportion of both CD4 positive and CD4 negative T cells [7]. Furthermore, B20.4.1.1 reduced clinical scores over a later time window (18–21 days) than bevacizumab (13–21 days, [7]). The later window strongly implicates angiogenesis in the effect of B20-4.1.1 since this is the period during which angiogenesis is detected (fig. 2). Therefore, B20.4.1.1 provides evidence for an impact of angiogenesis during EAE, and a direct role for VEGF. These data suggest that angiogenesis is linked to peripheral T cell activation, but suggest a disconnection between angiogenesis and T cell recruitment to the diseased CNS. This is likely given that T cell recruitment occurs across postcapillary venules, while angiogenesis tends to generate smaller, capillary sized microvessels that play a limited role in this recruitment. Further evidence for the link between angiogenesis and peripheral T cell activation (as opposed to CNS recruitment) comes from the lack of an effect on proliferation or cytokine production when isolated T cells were treated with B20.4.1.1 (fig. 8). The effects of B20.4.1.1 therefore depend on administration to the whole animal, and to linked processes that operate in vivo, such as suppressed angiogenesis.

### Effect of K(1-3) on EAE and Comparison to B20.4.1.1

Further evidence that angiogenesis plays a role in exacerbating EAE comes from data with the first three kringle domains of angiostatin. Angiostatin and K(1-3) are representative of direct angiogenesis inhibitors that function through effects on vascular endothelial cells to inhibit new blood vessel formation [25]. This study is the first to explore the effect of direct angiogenesis inhibitors such as K(1-3) on EAE. Angiostatin is a cleavage product of plasminogen that contains the first four kringle domains (loop structures containing about 80 amino acids). It has been shown to inhibit angiogenesis by direct targeting of vascular endothelial cells, although the precise mechanism varies depending on the study. Mechanisms include binding to putative endothelial receptors (ATPase, angiomotin, [12]–[14]), cell cycle arrest at the G2/M transition [15] and induction of endothelial apoptosis [16]–[18]. In our hands, K(1-3) had a biphasic effect on angiogenesis. At day 14, it caused an unexpected increase in vessel counts, however these were found in white matter rather than gray matter. By day 21, this transient increase in white matter counts had returned to levels seen in untreated mice, and the increase in gray matter counts had been suppressed (fig. 2B). This unexpected effect of K(1-3) suggests that it may transiently stimulate new vessels or arrest them at any early stage before angiogenesis is fully established. The reduction in clinical scores after treatment with K(1-3) occurs between days 18–21, which parallels B20.4.1.1 and reinforces the linkage between EAE and angiogenesis (fig. 1). Both agents failed to significantly alter T cell infiltration into the spinal cord, despite their ability to suppress angiogenesis (fig. 6) which reinforces the contrast to bevacizumab and the likelihood that bevacizumab inhibits spinal cord recruitment through mechanisms unrelated to angiogenesis. The small increase in the relative proportion of CD4 negative T cells in the K(1-3) group (fig. 6C) is of questionable biological significance. However K(1-3) and B20.4.1.1 did not have identical effects. K(1-3) had no effect on vascular permeability and did not reduce peripheral T cell proliferation (figs. 4,5). However, it had an identical impact on IL-17 release by peripheral lymphoid cells (fig. 7) emphasizing the link between angiogenesis suppression and this particular T cell response. Once again, K(1-3) had no effect beyond treatment of the whole animal. Proliferation and cytokine responses in isolated T cells did not respond to treatment with K(1-3) (fig. 8). How angiogenesis affects peripheral T cell responses is conjectural, but one possibility is that new blood vessels allow increased trafficking of antigen presenting cells from the central nervous system (CNS) to peripheral lymph nodes. Another possibility is that CNS antigens are transported directly along perivascular spaces to peripheral lymphatics, and that antigen delivery is enhanced by a greater density of blood vessels [29]. The different impact of B20-4.1.1 and K(1-3) on vascular permeability is also of interest. Permeability is generally modulated at the level of postcapillary venules in the CNS. VEGF has multiple effects apart from being pro-angiogenic that include increases in vascular permeability in the CNS. It is therefore not surprising that permeability is reduced by treatment with B20-4.1.1 (fig. 4). The lack of effect of K(1-3) on permeability is also not surprising if one considers that angiogenesis mostly increases capillaries rather than postcapillary venules, so that reducing the density of capillaries is likely to have little impact on permeability. This suggests that the impact of B20-4.1.1 on permeability results from reduced binding of VEGF to postcapillary venules, rather than through suppressed angiogenesis. These data also show an inverse relationship between permeability and vessel counts at day 14 in the B20.4.1.1 treated mice, but not in the K(1-3) treated group. At day 21, there is little relationship between these parameters for either treatment (fig. 4B,C). The inverse relationship seen in the B20.4.1.1 treated mice at day 14 is not seen in untreated EAE [6]. We speculate that the removal of VEGF by treatment with B20.4.1.1 tilts the balance in favor of factors that suppress permeability. This would explain the lack of effect with K(1-3). The relationship to vessel density suggests that pre-existing vessels produce these inhibitory factors. Why this relationship is lost at day 21 (when angiogenesis is suppressed) is unclear. Finally, K(1-3) or angiostatin may have clinical advantages over VEGF blocking agents such as bevacizumab, making it a more attractive candidate for treating Multiple Sclerosis. Long-term blockade of VEGF by bevacizumab reduces levels of nitric oxide leading to cardiovascular complications such as hypertension [30], . However, angiostatin appears to be relatively well tolerated when administered in clinical trials, perhaps because it acts principally by targeting activated vascular endothelial cells [32], [33]. This might be a key factor in determining its acceptability as a clinical treatment.

## Conclusions

This study demonstrates that murine EAE responds to treatment with both a direct and an indirect inhibitor of angiogenesis. Clinical scores reduce in parallel to the suppression of angiogenesis by both agents in the spinal cord. This shared effect from two agents with different mechanisms of action (VEGF binding by B20-4.1.1 and vascular endothelial inhibition by K(1-3)) is likely to produce secondary effects on peripheral immune activation. Both agents affected aspects of peripheral T cell activation such as Th17 cytokine expression, however neither agent affected these responses when applied to T cells in isolation or produced major changes in T cell infiltration into the spinal cord. This study therefore adds to the evidence that angiogenesis plays a pathogenic role in EAE and by extension MS. Angiogenesis inhibitors like K(1-3) or angiostatin with minimal side effects (in contrast to bevacizumab, the drug most closely related to B20-4.1.1) may represent a therapeutic option for the more effective treatment of MS. Further studies are warranted to delineate the complex effects of both agents and to explore the regulation and impact of angiogenesis in inflammatory autoimmune diseases of the CNS.
